# Mental arithmetic modulates temporal variabilities of finger-tapping tasks in a tempo-dependent manner

**DOI:** 10.7717/peerj.13944

**Published:** 2022-08-25

**Authors:** Shun Irie, Yoshiteru Watanabe, Atsumichi Tachibana, Nobuhiro Sakata

**Affiliations:** 1 Division for Smart Healthcare Research, Dokkyo Medical University, Mibu-machi, Tochigi, Japan; 2 Major of Physical Therapy, Department of Rehabilitation, School of Health Sciences, Tokyo University of Technology, Ota-ku, Tokyo, Japan; 3 Department of Anatomy, Dokkyo Medical University, Mibu-machi, Tochigi, Japan; 4 Center for Information & Communication Technology, Dokkyo Medical University, Mibu-machi, Tochigi, Japan

**Keywords:** Dual-task paradigm, Cognitive function, Mental arithmetic, Finger-tapping

## Abstract

**Background:**

Several psychiatric diseases impair temporal processing. Temporal processing is thought to be based on two domains: supra-second intervals and sub-second intervals. Studies show that temporal processing in sub-second intervals is mainly an automated process. However, the brain functions involved in temporal processing at each time scale remain unclear. We hypothesized that temporal processing in supra-second intervals requires several brain areas, such as the ventrolateral prefrontal cortex, intraparietal sulcus (IPS), and inferior parietal lobe, corresponding to various cognitions in a time scale-dependent manner. We focused on a dual-task paradigm (DTP) involving simultaneous performance of cognitive and motor tasks, which is an effective method for screening psychomotor functions; we then designed a DTP comprising finger tapping at various tempi as the temporal processing task and two cognitive tasks (mental arithmetic and reading) that might affect temporal processing. We hoped to determine whether task-dependent interferences on temporal processing in supra-second intervals differed depending on the cognitive tasks involved.

**Methods:**

The study included 30 participants with no history of neuromuscular disorders. Participants were asked to perform a DTP involving right index finger tapping at varying tempi (0.33, 0.5, 1, 2, 3, and 4 s inter-tapping intervals). Cognitive tasks comprised mental arithmetic (MA) involving three-digit addition, mental reading (MR) of three- to four-digit numbers, and a control (CTL) task without any cognitive loading. For comparison between tasks, we calculated the SDs of the inter-tapping intervals. Participants’ MA abilities in the three-digit addition task were evaluated.

**Results:**

The MA and MR tasks significantly increased the SDs of the inter-tapping intervals compared to those of the CTL task in 2–3 s and 3–4 s for the MA and MR tasks, respectively. Furthermore, SD peaks in the finger-tapping tasks involving MA were normalized by those in the CTL task, which were moderately correlated with the participants’ MA ability (*r* = 0.462, *P* = 0.010).

**Discussion:**

Our results established that DTP involving the temporal coordination of finger-tapping and cognitive tasks increased temporal variability in a task- and tempo-dependent manner. Based on the behavioral aspects, we believe that these modulations of temporal variability might result from the interaction between finger function, arithmetic processing, and temporal processing, especially during the “pre-semantic period”. Our findings may help in understanding the temporal processing deficits in various disorders such as dementia, Parkinson’s disease, and autism.

## Introduction

Temporal processing is one of the cognitive domains related to temporal dimensions. It is often impaired by several psychiatric diseases, such as a bipolar disorder, Parkinson’s disease, dementia, and autism (Folstein, Folstein & McHugh, 1975; Kuhs et al., 1991; Kjelgaard & Tager-Flusberg, 2001; Mahlberg et al., 2008; Casassus et al., 2019; Buciuc et al., 2021; Terao et al., 2021; Yeung et al., 2021). Temporal processing is sometimes divided into two sub-categories: sub-second intervals and supra-second intervals (c.f. Pöppel, 2009). Furthermore, the functional localizations are believed to differ depending on the time scale (Grondin, 2012). For sub-second intervals, the minimum window for temporal processing is approximately 40 ms, and it is considered an automatic process (Lewis & Miall, 2003; Pöppel, 2009). Indeed, Wiener, Turkeltaub & Coslett (2010) demonstrated that the sub-cortical networks, such as the basal ganglia and the cerebellum, are dominantly involved in the temporal processing in sub-second intervals. Additionally, some brain areas associated with motor execution and regulation systems are also involved in temporal processing in sub-second intervals, such as the premotor cortex, supplementary motor area (SMA), and the dorsolateral prefrontal cortex (DLPFC) (Ivry & Keele, 1989; Lewis & Miall, 2002).

However, the temporal processing in supra-second intervals is considered to be regulated by a cognitively controlled system (Lewis & Miall, 2003). In contrast to the temporal processing in sub-second intervals, supra-second processing primarily recruits cortical structures. The cortical areas related to attention and working memory, such as the ventrolateral prefrontal cortex, the intraparietal sulcus (IPS), and the inferior parietal lobe, are known to be involved in supra-second processing. Additionally, some brain areas are involved in both sub-and supra-second processing, such as the SMA, the DLPFC, and prefrontal cortex (for a review, see Lewis & Miall, 2003). However, the contributions of cognitive domains such as language, arithmetic, and memory to temporal processing at various time scales, including sub- and supra-second intervals, remain unclear. Additionally, we must consider the possibility that temporal processing in supra-second intervals is further divided into other sub-categories (c.f. Pöppel, 2009).

Based on the aspects mentioned above, we focused on the dual-task paradigm (DTP) procedure, which involves the simultaneous performance of motor and cognitive tasks in which the cognitive load interferes with motor performance. The amount of interference in the DTP indicates the functional overlap across two different tasks or the amounts of cognitive loads on the central executive system (D’Esposito et al., 1995; Yogev-Seligmann, Hausdorff & Giladi, 2008). Furthermore, a DTP is a quick and straightforward assessment tool that can be applied in clinical contexts (Springer et al., 2006; Doi et al., 2013; Ghai, Ghai & Effenberg, 2017; Tait et al., 2017; Lo et al., 2020; Mancioppi et al., 2021). Therefore, we assessed the mechanisms associated with temporal processing at various time scales using a DTP designed for clinical applications, as they pertain to treating diseases associated with temporal processing disorders.

As components of the DTP, we focused on finger-tapping and mental arithmetic (MA) tasks because MA tasks are believed to interfere with finger-tapping tasks (Hiscock et al., 1989; Fraser, Li & Penhune, 2010; Sommervoll, Ettema & Vereijken, 2011; Samulski et al., 2020). Interference between finger-tapping, temporal processing, and arithmetic tasks is thought to result from finger counting strategies during mental calculations (Hayashi et al., 2013; Soylu & Newman, 2016; Soylu, Lester & Newman, 2018). Additionally, finger tapping at an established tempo has been frequently used as a DTP for evaluating motor tasks in experimental and clinical settings (Hiscock et al., 1989; Fraser, Li & Penhune, 2010; Sommervoll, Ettema & Vereijken, 2011; Samulski et al., 2020). Finger tapping at an established tempo is likely regulated by some of the brain areas responsible for time perception (Zanto et al., 2019). Recently, Bååth, Tjøstheim & Lingonblad (2016) reported that the working memory task (N-back test) interferes with the temporal variability of inter-tap intervals (ITIs) and that such interference is only observed for slower ITIs. The neural system associated with finger tapping at an exclusively slower tempo and the working memory are believed to functionally overlap.

Therefore, based on behavioral aspects, MA-induced interferences result from different neural mechanisms compared to the N-back test involving finger tapping. According to Grondin’s (2012) theory, the tempo at which finger tapping faces significant interference from cognitive loads may differ across cognitive tasks conducted concurrently with finger tapping.

Thus, we hypothesized that the interference of cognitive tasks on the performance of finger-tapping tasks in the DTP is modulated in a tempo- and task-dependent manner. Unlike previous studies that used the DTP of finger tapping and one cognitive task, in this study, we assessed temporal disturbance during the DTP with finger tapping and cognitive tasks from two perspectives: the time scale (tempo of finger tapping) and the type of cognitive task (MA and mental reading (MR)). The MR task was set as the control task for comparison with the DTP, using the MA task to distinguish the effect of numeric reading from that of calculation on the temporal coordination of finger tapping (Taler & Jarema, 2004; Lo et al., 2020).

## Materials & Methods

### Participants

Thirty participants (eight women and 22 men; aged 21–61 years) with no history of neuromuscular disorders participated in the experiments (see ‘Experimental Procedure’). All participants except one were right-side dominant (29/30 participants) in accordance with the Edinburgh inventory (Oldfield, 1971).

**Figure 1:**
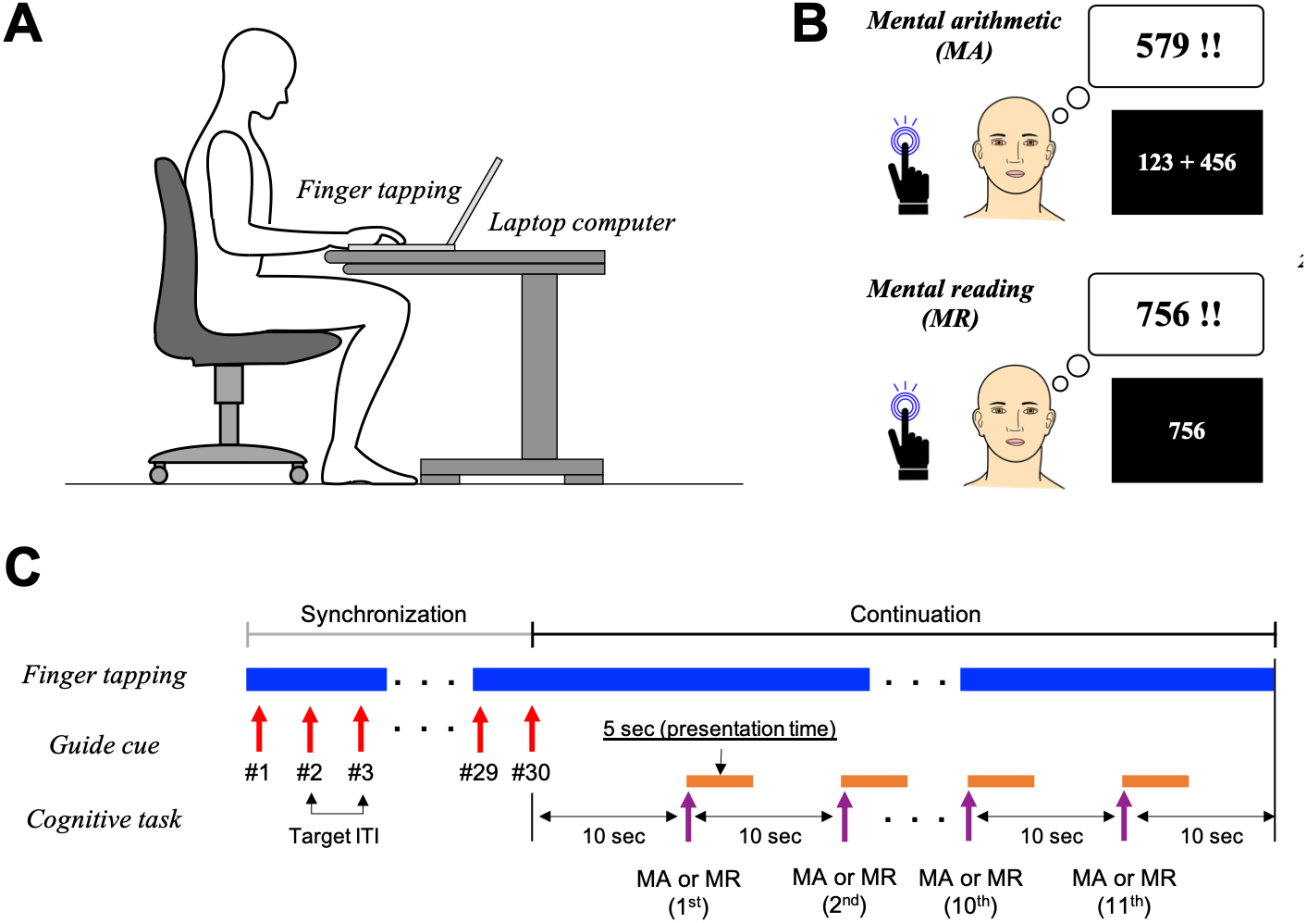
Experimental procedures. (A) The participants sat on a chair in front of a desk. A laptop computer was placed on the desk. (B) In the two task trials during finger tapping in the mental arithmetic (MA) task, participants were asked to conduct a three-digit addition mentally. In the mental reading (MR) task, participants were asked to recall the three- to four-digit numbers ranging from 201–1997. (C) During the synchronization trial, the participants performed finger tapping with fixed tempo using visual cues presented on the screen (red circle). The cues were presented for 200 ms with several inter-tapping intervals (ITI) ranging 0.33–4 s and repeated 30 times. Immediately after the synchronization trial, participants continued to perform finger tapping. Ten seconds after the synchronization trial, the task indication (*e.g.,* three-digit additions for MA or three- to four-digit numbers for MR tasks) was presented at the center of the screen. The presentation time and inter-stimulus interval (ISI) of the cognitive tasks (MA and MR) were set to 5 and 10 s, respectively.

To reduce the risk of COVID-19 infection under the emergency declaration by the Japanese government, the experiments were conducted remotely using a custom-made web application deployed on Google Firebase and coded using Hyper Text Markup Language (HTML) and JavaScript^®^ under the jsPsych framework (De Leeuw, 2015; Provenza et al., 2021). Informed consent was obtained using a web-meeting system (Teams, Microsoft, WA), and written informed consent was obtained via post after the experiments. In accordance with the Declaration of Helsinki, the study protocol was approved by the Research Ethics Committee of Dokkyo Medical University (approval No. 2021-011) and the Tokyo University of Technology (approval No. E20HS-038). During the experiment, participants were asked to sit in a chair in a quiet room and place their right index finger on the Enter key of the keyboards provided (Fig. 1A).

### Finger-tapping task

In this study, we defined the inter-onset interval (IOI) as the target tempo of the finger-tapping task; ITI denotes the finger-tapping interval obtained from participants. The participants were asked to perform the right index finger-tapping task involving varying IOIs of 0.33, 0.5, 1.0, 2.0, 3.0, and 4.0 s simultaneously with the cognitive tasks. However, the participants were asked to refrain from counting the beats by moving their bodies. Prior to the experiment sessions, all participants conducted the finger-tapping task at a constant tempo using visual cues presented on the screen as a primer (synchronization trial; Fig. 1C). The visual timing cues were red-colored circles (*φ* = 2.2 cm) and were presented for 200 ms in all IOIs (Madison et al., 2013). The participants were asked to tap the Enter key in synchrony with the visual cue to determine the finger-tapping tempo. The visual cues were presented 30 times with each IOI.

After the synchronization trial, the participants were asked to continue finger-tapping without visual cues with as fixed a tempo as possible (Fig. 1C). Ten seconds later, the task trials started, minimizing the temporal variability of ITI derived from non-cognitive factors (synchronization-continuation paradigm) (Jantzen, Steinberg & Kelso, 2004; Qi et al., 2019).

For the task trials, the participants were asked to focus their eyes at the center of the screen while simultaneously performing cognitive tasks and finger tapping with the same tempo as in the synchronization trial, although without visual timing cues. The finger-tapping task lasted 120 s (11 cognitive trials; see ‘Cognitive Tasks’) (Bååth, Tjøstheim & Lingonblad, 2016).

### Cognitive tasks

In the experiment sessions, the participants performed three cognitive tasks: MA, MR, and control (CTL) tasks. All tasks comprised 11 trials performed in 10 s cycles.

For the MA tasks, an arithmetic task involving triple-digit addition (*e.g.,* 123 + 456) was presented at the center of the screen (Fig. 1A). Triple-digit addition was chosen because double- or single-digit addition is relatively easy. Such addition often involves retrieval as well as calculation, making it unsuitable for research related to calculation functions (Thevenot et al., 2013). The presentation time and inter-stimulus interval (ISI) were set to 5.0 s and 10.0 s, respectively (Fig. 1C). In the MA task session, the participants were asked to calculate the answer to the problem presented on the screen as quickly as possible; however, they were asked not to vocalize their answers or calculation processes to block the effects of vocalization (Van Rooteselaar, Beke & Gonzalez, 2020).

For the MR task, a three- or four-digit number ranging from 201 to 1997—corresponding to the ranges of possible answers to the MA tasks—was presented on the screen (Fig. 1B). The presentation time and ISI were the same as those in the MA task. In the MR task sessions, the participants were asked to recall the numbers presented on the screen without any vocalizations. Furthermore, to block the effects of local dialects and pronunciations of the numerical digits, participants were asked to mentally recall the numbers in the standard Japanese language (*i.e.,* 1234 is pronounced “*sen-hyaku-sanjuu-yon*”) (Lewis et al., 2020). For the MA and MR tasks, we verbally confirmed after task completion that participants had conducted the experimental tasks.

The participants were asked to continue the finger-tapping task without any cues or cognitive tasks for the CTL task.

### Experimental procedure

Eighteen finger-tapping sessions with three cognitive tasks (task trials) under six different IOIs (experimental sessions) were performed by 30 healthy participants. In general, the participants were asked to conduct finger tapping simultaneously with the cognitive tasks comprising MA, MR, or CTL in the same IOI in random order. The order of the target IOIs was also randomized. The participants took a one-minute break between the finger-tapping sessions.

After the synchronization-continuation task, the participants performed a MA task by typing the answers at the end of the experiments. In this task, the participants solved the arithmetic problems presented on the screen by using the digit keys of the keyboard. The presentation time, ISI, and difficulty (triple-digit addition) were the same as those in the MA task.

### Data analysis

The primary outcomes were the standard deviations (SDs) of ITIs during the task trials (MA, MR, and CTL) in different target IOIs. For inter-participant comparisons, the SDs of ITIs during the MA and MR tasks were normalized to those during the CTL task (modulatory effects).

The accuracy and response times of the arithmetic tasks were also calculated to evaluate the MA function. Accuracy was defined as the correct answer rate, which involved the number of complete answers for eleven problems divided by the total number of problems.

### Statistical analysis

For the means, SDs, and modulatory effects, a repeated-measures (RM) ANOVA and Holm post-hoc test (paired *t*-test with Holm’s correction) were conducted. For RM-ANOVA, the dependent variable was set to the means, SDs, or modulatory effects of the ITIs. The independent variables were assigned to both the IOI and cognitive tasks. For comparison of the peak IOI of the modulatory effect between the MA and MR tasks, the Wilcoxon signed rank test was also performed.

The correlation coefficients (*r*) were calculated to assess the relationship between the maximum modulatory effects for each participant induced by the MA and MR tasks. Furthermore, the *t*-value and *p*-value for the null hypothesis (*r* = 0) were calculated.

The group data are expressed as the mean ± SE unless otherwise noted. Statistical analyses were performed using R version 4.1.0 (R Core Team 2021. R: Language and Environment for Statistical Computing. R Foundation for Statistical Computing, Vienna, Austria). Effect size indices were determined using Cohen’s *f* for the RM-ANOVA using G*power (version 3.1.9.6; Heinrich Heine University, Düsseldorf, Düsseldorf, Germany) (Faul et al., 2007; Cohen, 2009).

## Results

### Effects of cognitive tasks on the means and SDs of ITIs

**Figure 2:**
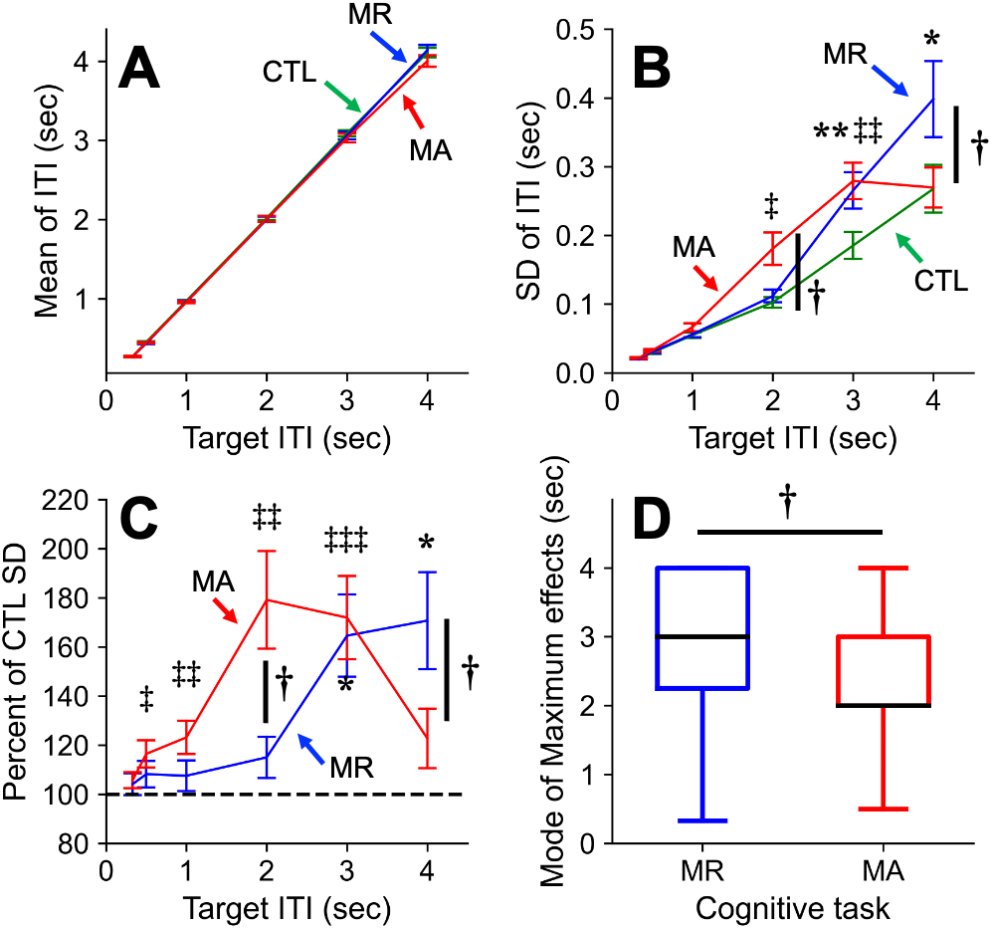
Main results of this study. Means (A), SDs (B), and the modulatory effects (C) of the ITIs in different target ITIs during control (CTL) (green lines), MA (red lines), and MR (blue lines) tasks are shown in the left and right panels, respectively. (D) Boxplots for the ITI which the maximum modulatory effects were observed. The error bars indicate the standard error of means. * *p* < 0.05; ** *p* < 0.01; (MR vs CTL); † *p* < 0.05 (MR vs MA); ‡, ‡‡, ‡‡‡ *p* < 0.05, 0.01, 0.001 (MA vs CTL) (Holm *post-hoc* test and the Wilcoxon signed rank test).

Figure 2 shows the means (A) and SDs (B) of ITIs under the three tasks for each IOI. For the ITIs, RM-ANOVA indicated a significant main effect within the factor of IOI; however, we did not observe a significant main effect within the tasks or an interaction between factors (IOI, *F*_5,145_ = 3300, *f* = 10.64, *P* < 0.001; Task, *F*_2,58_ = 2.646, *f* = 0.302, *P* = 0.080; target IOI × Task, *F*_10,290_ = 1.473, *f* = 0.225, *P* = 0.149). In contrast, the SDs of ITIs were significantly greater than those under the CTL task, while the IOIs were 2–3 and 3–4 s for the MA and MR tasks, respectively. Additionally, we observed significant differences in those under the MA and MR tasks, while the IOI was 2 and 4 s, respectively (IOI, *F*_5,145_ = 66.27, *f* = 1.512, *P* < 0.001; Task, *F*_2,58_ = 8.672, *f* = 0.547, *P* < 0.001; IOI × Task, *F*_10,290_ = 6.879, *f* = 0.487, *P* < 0.001, post-hoc Holm test; *P* < 0.05). These are summarized in Table 1.

**Table 1:** Results of two-way ANOVA for each dependent variables.

*Factor*	*df*	*F*	*Cohen’s f*	*P*
Dependent variable: Mean ITI (s)				
IOI	5	3,300	10.64	<0.001^*^
Task	2	2.646	0.302	0.080
IOI × Task	10	1.473	0.225	0.149
Dependent variable: SD ITI (s)				
IOI	5	66.27	1.512	<0.001^*^
Task	2	8.672	0.547	<0.001^*^
IOI × Task	10	6.879	0.487	<0.001^*^
Dependent variable: modulatory effects (% of CTL ITI)				
IOI	5	6.818	0.485	<0.001^*^
Task	2	19.44	0.819	<0.001^*^
IOI × Task	10	6.517	0.474	<0.001^*^

**Notes.**

* *P* < 0.001.

### Tempo effects of the modulatory effects under the MA and MR tasks compared to CTL

Figure 2C shows the means of the modulatory effects under the MA and MR tasks for each IOI. The mean modulatory effects were significantly greater than those under the CTL task, while the IOIs were 0.5–3 and 3–4 s for the MA and MR tasks, respectively (IOI, *F*_5,145_ = 6.818, *f* = 0.485, *P* < 0.001; Task, *F*_2,58_ = 19.44, *f* = 0.819, *P* < 0.001; target IOI × Task, *F*_10,290_ = 6.517, *f* = 0.474, *P* < 0.001, *post-hoc* Holm test; *P* < 0.05, Table 1). Additionally, the differences between the modulatory effects under the MR and MA tasks at 2 and 4 s, respectively, reached a significant level (*post-hoc* Holm test; *P* < 0.05).

Figure 2D shows the modes of IOIs for the maximum modulatory effects under the MA and MR tasks. From the Wilcoxon signed rank test, the mode of the IOI with the maximum modulatory effects in the MR task was significantly longer than that for the MA task (Median, MR: 3 s, MA: 2 s, Wilcoxon signed rank test, *V* = 132, *P* = 0.044).

### Correlation analysis between the MA-induced modulation and arithmetic ability

**Figure 3:**
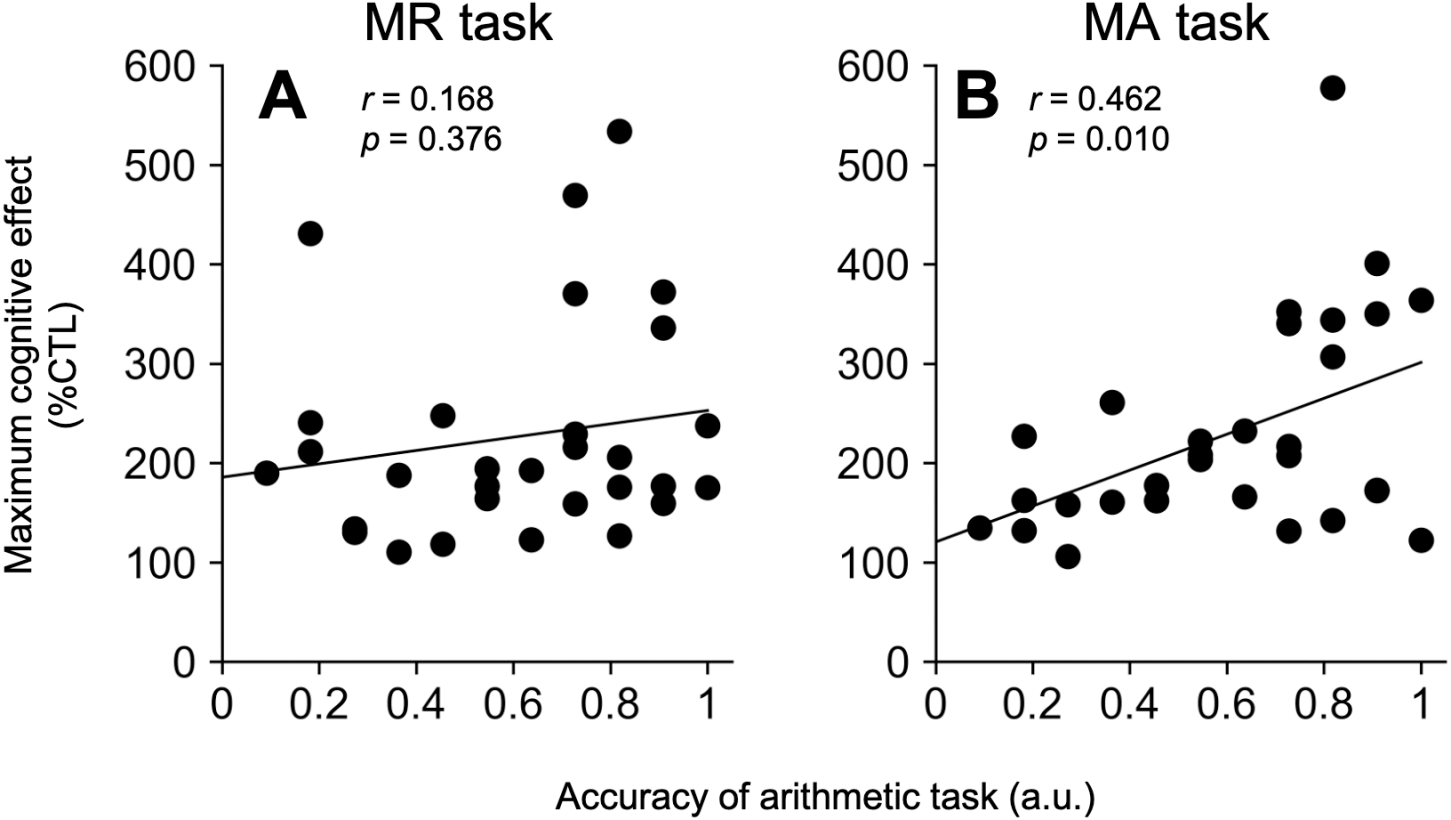
Relationship between maximum modulatory effects and mental arithmetic abilities. The pairs of the correct answer rate of the MA task and maximum modulatory effects are shown using scatter plots in the MR (A) and MA (B) tasks. The solid lines indicate regression lines.

We calculated the means of the correct answer rate and response time for the MA task performed after all DTP trials (correct answer rate: 0.61 ± 0.05, response time: 4.65 ± 0.15 s). Figure 3 shows scatter plots of the accuracy of MA answers and the maximum modulatory effects under the MR (A) and MA (B) tasks. During the MA task, the correlation coefficients between both pairs of factors (modulatory effects *vs.* arithmetic correct answer rate) reached a significant level, but not under the MR task (MR, *r* = 0.168 (95% CI −0.205–0.498), *t*_28_ = 0.900, *P* = 0.376, MA, *r* = 0.462 (95% CI [0.122–0.705]), *t*_28_ = 2.755, *P* = 0.010, Figs. 3A–3B).

## Discussion

This study demonstrated that the cognitive tasks increased the temporal variabilities during right index finger tapping, in a task-specific manner (MA: 0.5–3, MR: 3–4 s). The conditions with IOIs of 2 s and 4 s showed significant differences between the MA and MR tasks. Furthermore, the magnitude of modulatory effects induced by the MA tasks positively correlated with the MA ability, thereby suggesting the existence of a functional overlap between finger motion, temporal processing, and/or the arithmetic functions.

### Possible mechanisms of temporal interference during finger-tapping tasks with cognitive tasks

Bååth, Tjøstheim & Lingonblad (2016) showed that the temporal variability of finger tapping with the working memory task (N-back) was larger while tapping at slower tempi (e.g., 2 and 3 s IOI). This is consistent with the temporal features of the finger tapping involving the MR task in this study (see Fig. 2B and 2C). In contrast, Holm et al. (2017) suggested that the temporal variability of finger tapping with a concurrent memory task was larger while tapping at the tempi of sub-second IOIs. Additionally, several reports suggested that the magnitudes of the cognitive load were related to the amount of interferences while the dual tasks were performed (Watanabe & Funahashi, 2014). Thus, the magnitude of the modulations on the temporal variability of the finger tapping may depend on a combination of tempi, cognitive tasks, and cognitive loads (Holm, Ullén & Madison, 2013; Holm et al., 2017).

For MA, the functional overlapping between finger movement and numeric calculation was supported by several studies (c.f. Berteletti & Booth, 2015; Berteletti & Booth, 2016; Barrocas et al., 2020). For single-digit addition, Jordan et al. (2008) reported that the strategy for solving addition during childhood mainly involves counting fingers, which would be replaced by the retrieval strategy at the adult stage. This result was consistent with several psychological and neuroimaging studies related to not only single but also two-digit arithmetic (Michaux et al., 2013; Crollen & Noël, 2015; Soylu & Newman, 2016; Soylu, Lester & Newman, 2018). In fact, the performances of arithmetic and/or finger tapping interfered with each other (Michaux et al., 2013; Crollen & Noël, 2015; Soylu, Lester & Newman, 2018). Interestingly, recent neuroimaging and transcranial magnetic stimulation (TMS) studies suggest that such interferences between arithmetic and finger motor functions subliminally exist during the adult stage, even though adults do not use finger-counting strategies to solve addition problems (Sato et al., 2007; Michaux et al., 2013; Soylu & Newman, 2016). Furthermore, neuroscientific studies also suggest that MA requires several neural substrates that are potentially related to finger movements, such as the primary motor cortex (M1), inferior frontal gyrus (IFG), the cerebellum, and the IPS (Colby & Goldberg, 1999; Critchley et al., 2000; Cantlon et al., 2006; Sato et al., 2007; Hawes et al., 2019; McDougle et al., 2022). However, these studies mainly support single- and/or double-digit arithmetic, and not three-digit arithmetic. In three-digit addition, the taxonomies of strategies for calculation are summarized into three categories: (1) stepwise (*e.g.,* 123 + 456 = 123 + 400 + 50 + 6), (2) split (e.g., 123 + 456 = (100 + 400) + (20 + 50) + (3+6)), and (3) compensation (e.g., 527 + 398 = 527 + 400 − 2), which suggest that three-digit addition could be solved through pure calculation processes and different strategies, unlike single- and/or double-digit addition (Fuson et al., 1997; Selter, 2001; Heinze, Marschick & Lipowsky, 2009; Csíkos, 2016). Thus, the tempo-dependent interferences of temporal coordination in this study were a result of calculation processes rather than retrieval processes. Considering the significant relationship between the magnitudes of interference in the finger-tapping for MA and the arithmetic abilities, the functional overlapping between arithmetic and finger functions might be involved with task-dependent interference in this study.

For the finger-tapping task, the task sequences in the synchronization and continuation of the finger-tapping task after turning off the rhythmic visual cues in this study are well known as the synchronization-continuation paradigm, which is closely related to time perception (Zanto et al., 2019). Importantly, distinct brain areas involved in regulating the temporal coordination of finger tapping may differ according to the tempo (Rao et al., 1997; Lewis & Miall, 2003; Jantzen, Steinberg & Kelso, 2004; Bååth, Tjøstheim & Lingonblad, 2016). We must consider the different mechanisms in the sub-second (<1 s), pre-semantic (<2–3 s), and longer periods (>3–4 s) (Lewis & Miall, 2003; Pöppel, 2009). The finger tapping in sub-second intervals mainly relied on automatic processing, whereas that in supra-second intervals, including pre-semantic and longer time range, required cognitive attention (Mitrani et al., 1977; Rammsayer, 1999; Lewis & Miall, 2003). Interestingly, the MA and MR tasks interfered with the temporal coordination of finger tapping in a different manner. The maximum interferences were mainly observed in the pre-semantic periods (<2–3 s) and later IOIs (>3–4 s) for the MA and MR tasks, respectively.

The pre-semantic period involved temporal segmentation based on automatic (pre-semantic) cognitive processes (c.f. Pöppel, 2009). The concept of the pre-semantic period was supported by various psychological studies on motor behavior, visual perception, memory, and speech fields (Pöppel, 1971; Getty, 1975; Kowal, O’Connell & Sabin, 1975; Kien & Kemp, 1994; Gomez et al., 1995; Ilg et al., 2008). For the MR task, the temporal variabilities were increased when the IOI was over 3 s, near the end of the pre-semantic period. Kien & Kemp (1994) reported that the temporal segmentations for reading sentences in German and Korean poems were 1.7 s and 2.24 s, respectively. Although there are no studies on Japanese speaking, to the best of our knowledge, our results in the MR tasks might reflect temporal segmentation for reading because Japanese has many grammatical similarities with Korean (Shibatani, 1990; Kien & Kemp, 1994). In contrast to the MR task, the interferences for temporal variabilities induced by the MA task were mainly observed only within the putative pre-semantic period. Kagerer et al. (2002) reported that patients with right hemispheric lesions were impaired in a temporal reproduction task with stimuli longer than 2–3 s, but they were not impaired for stimuli of less than 2–3 s. Therefore, the temporal interferences induced by the MA task could result from the functional overlapping between the cortical and sub-cortical regions related to arithmetic function and temporal cognition within the pre-semantic period, such as SMA, putamen, thalamus, IFG, IPS, and the cerebellum (Ivry & Keele, 1989; Rao et al., 1997; Jäncke et al., 2000; Jantzen, Steinberg & Kelso, 2004; Cantlon et al., 2006; Hayashi et al., 2013; Hawes et al., 2019; McDougle et al., 2022). Moreover, Hayashi et al. (2013) demonstrated the interaction between numericity and time perception in the prefrontal cortex and the parietal cortex. These brain areas might also be involved in the functional overlap between finger tapping and MA.

In this section, we have discussed the possible mechanisms on the assumption that the interferences during the DTP were due to the functional overlap between two tasks, called the “Overlapping hypothesis” (Klingberg & Roland, 1997; Klingberg, 1998; Nijboer et al., 2014). However, as another possible mechanism, we should also consider the effects of the central executive system, which is independent of the center of task commands (D’Esposito et al., 1995). D’Esposito et al. (1995) demonstrated that the activation of the prefrontal cortex was solely observed while participants performed the dual task, not single tasks. In this theory, each task composing the dual task requires the common resources in the central executive system. Thus, while performing the dual task, the excessive demand from each task on the common resources results in a decrease in the quality of both tasks. This is supported by other reports involving the DLPFC, inferior frontal junction, superior medial frontal cortex, and insula (Just et al., 2001; Just, Keller & Cynkar, 2008; Tombu et al., 2011; Tachibana et al., 2012). Generally, the temporal reproduction in longer time (including the finger tapping with static tempi) requires more consciousness compared to that in shorter time (Pöppel, 2009). Additionally, the cognitive loads in the MA task would be larger than those in the MR task. Based on this hypothesis, more cognitive resources were required in the finger tapping involving the MA task under 4 s IOIs compared to those involving the MR task, which is not consistent with the results of this study. Thus, it is likely that the interferences on temporal coordination of finger tapping with the MA tasks were a result of functional overlapping rather than the central executive system.

### Functional implications

In this study, we found that the magnitudes of temporal interferences during the DTP with finger tapping and cognitive tasks were dependent on both types of cognitive tasks and finger tapping IOIs. In particular, MA caused interference with a 2–3 s IOI, MR with a 3–4 s IOI. The current results potentially contribute to the understanding of arithmetic, language processing, and temporal processing. Clinically, the deficiencies of these functions were frequently observed in patients with various psychiatric conditions such as dementia, depression, Parkinson’s disease, and autism (Folstein, Folstein & McHugh, 1975; Kjelgaard & Tager-Flusberg, 2001; Casassus et al., 2019; Buciuc et al., 2021; Terao et al., 2021; Yeung et al., 2021). Furthermore, the cognitive impairments in these disorders are known to appear in complex combinations rather than in a fixed pattern (American Psychiatric Association, 2013). In this study, we examined the effects of two combinations with finger tapping (temporal processing) and the MA/MR tasks. Although this study was conducted on healthy participants, the comparison of these tempo- and task-dependent interferences between healthy participants and patients with various neurological deficits may result in the understanding of the origins of complex symptoms in such diseases, which could be advanced to a biological marker for pharmacotherapy (Davison et al., 2011; Nathan et al., 2013). Therefore, further studies are required involving behavioral and physiological techniques among healthy individuals, as well as among patients.

### Limitations

This study has some limitations. First, the cognitive loads of the MA in triple-digit addition are higher than those of the MR task. Therefore, it is difficult to exclude the possibility that the modulatory effects in MA tasks may have been derived from the magnitude of cognitive loads rather than the modalities of cognitive tasks (Bijarsari, 2021). However, as described above, the interferences on finger-tapping were observed in those with higher arithmetic abilities, which is the opposite of the effect reported in previous studies (Springer et al., 2006; Lo et al., 2020; Mancioppi et al., 2021). Notably, the modulatory effect induced by the MR task in 4 s IOI was greater than that generated by the MA task. However, the cognitive loads in MR seem lower than those in the MA tasks. Hence, we considered that these effects were likely tempo- and task-dependent in the MA and MR tasks rather than cognitive load-dependent. Additionally, we could not confirm that participants correctly completed the cognitive tasks because we monitored the participants’ actions remotely. Brain imaging techniques could visualize the brain activities related to the MA and MR separately (Tachibana et al., 2012). Thus, further studies using brain imaging techniques would also enable us to assess whether participants correctly conducted the cognitive tasks.

Second, we must consider the effects of the processing time for the cognitive tasks on these interferences. Previous studies suggested that the time segmentation for the reading task was approximately 2–3 s (c.f., Pöppel, 2009). In contrast, the MA tasks in this study required 4.65 s on average. Importantly, this processing time included the reaction time and traveling time of the finger to the numeric keys, not only purely calculation time. Considering this movement-related time in the MA task alone, the calculation time of triple-digit addition should be approximately 4 s. Therefore, reduced interference on the temporal coordination in the MA task might result in consistency between the calculation time and the ITI of finger tapping.

The third limitation is that finger tapping with each cognitive task was performed as distinct trials. Thus, the differences in the baseline ITI between the tasks may have affected the modulation effects (Bååth, Tjøstheim & Lingonblad, 2016). All experimental tasks were conducted using the synchronization-continuation paradigm to minimize these differences, and there were no significant differences across the tasks in each IOI (see Fig. 2). Therefore, the effects of baseline ITI in each task were minimized in our experiment.

Finally, the functional localization of the tempo- and task-dependent interference on temporal coordination remains unclear. This is because of the difficulty in detecting brain activities using functional magnetic resonance imaging (fMRI) while the finger tapping is executed with a tempo of 1–2 s IOI due to the lower signal–noise ratio (Rao et al., 1996; Rao et al., 1997). However, recent studies have suggested that non-invasive brain stimulation (NIBS) techniques, such as transcranial direct current stimulation (tDCS) and repetitive transcranial magnetic stimulation (rTMS), can induce a virtual lesion in the focused brain area. This method can efficiently detect functional localization, even if the brain regions are subliminally activated during the tasks (Porciello et al., 2014; Hartwigsen, 2015; Rothwell, 2018). Additionally, morphological studies involving stroke and/or brain injury patients will help us understand the functional localization of this phenomenon (Kagerer et al., 2002). Therefore, we believe that combining brain imaging techniques and NIBS among healthy individuals as well as patients can reveal the neural mechanisms in detail, including functional representations of the entire brain.

## Conclusions

Our data show that cognitive tasks increase temporal variability in finger-tapping tasks in a tempo- and task-dependent manner. The effects on temporal variability were observed during finger tapping with 2–3 s IOIs in the MA task, which is consistent with the pre-semantic period suggested by Pöppel (2009). Moreover, the magnitude of interferences involving the MA tasks, and not the MR tasks, was moderately correlated with MA abilities. However, these interferences induced by the MR task were linearly increased when the IOI was increased. These results suggest the existence of common cognitive processes between arithmetic and temporal processing during the pre-semantic period. However, further studies are required to determine this phenomenon’s underlying neural mechanisms.

##  Supplemental Information

10.7717/peerj.13944/supp-1 Data S1 Raw data The datasets of all parameters. The sheet names are associated with the number of Figures.

## Additional Information and Declarations

### Data Availability

The following information was supplied regarding data availability: The raw data is available in the Supplemental File.
